# Glutamine enhances pneumococcal growth under methionine semi-starvation by elevating intracellular pH

**DOI:** 10.3389/fmicb.2024.1430038

**Published:** 2024-07-09

**Authors:** Chengwang Zhang, Juncheng Liu, Xiaohui Liu, Yueyu Xu, Qingxiu Gan, Qinqian Cheng, Weiping Liu, Xiangmin Gao, Songquan Wu

**Affiliations:** ^1^Department of Basic Medical Science, School of Medicine, Lishui University, Lishui, Zhejiang, China; ^2^National Protein Science Facility, Tsinghua University, Beijing, China; ^3^School of Life Sciences, Tsinghua University, Beijing, China

**Keywords:** *Streptococcus pneumoniae*, methionine, semi-starvation, glutamine, growth, intracellular pH

## Abstract

**Introduction:**

Bacteria frequently encounter nutrient limitation in nature. The ability of living in this nutrient shortage environment is vital for bacteria to preserve their population and important for some pathogenic bacteria to cause infectious diseases. Usually, we study how bacteria survive after nutrient depletion, a total starvation condition when bacteria almost cease growth and try to survive. However, nutrient limitation may not always lead to total starvation.

**Methods:**

Bacterial adaptation to nutrient shortage was studied by determining bacterial growth curves, intracellular pH, intracellular amino acid contents, gene transcription, protein expression, enzyme activity, and translation and replication activities.

**Results:**

No exogenous supply of methionine results in growth attenuation of Streptococcus pneumoniae, a human pathogen. In this paper, we refer to this inhibited growth state between ceased growth under total starvation and full-speed growth with full nutrients as semi-starvation. Similar to total starvation, methionine semi-starvation also leads to intracellular acidification. Surprisingly, it is intracellular acidification but not insufficient methionine synthesis that causes growth attenuation under methionine semi-starvation. With excessive glutamine supply in the medium, intracellular methionine level was not changed, while bacterial intracellular pH was elevated to ~ 7.6 (the optimal intracellular pH for pneumococcal growth) by glutamine deamination, and bacterial growth under semi-starvation was restored fully. Our data suggest that intracellular acidification decreases translation level and glutamine supply increases intracellular pH to restore translation level, thus restoring bacterial growth.

**Discussion:**

This growth with intracellular pH adjustment by glutamine is a novel strategy we found for bacterial adaptation to nutrient shortage, which may provide new drug targets to inhibit growth of pathogenic bacteria under semi-starvation.

## Introduction

In nature, nutrient starvation is a common condition that bacteria encounter in their whole lives. Usually, we describe nutrient starvation as depletion of certain nutrient(s), in which state bacteria grow extremely slowly (Gray et al., 2019). However, between the nearly stopped growth under total starvation and full growth with sufficient nutrients, there exists an intermediate state that bacteria have an obvious growth but its growth is inhibited. Growth of *Streptococcus pneumoniae* in chemically defined medium (CDM) without methionine supply is a good example to exhibit this intermediate state. *S. pneumoniae* is an opportunistic human pathogen that colonizes in the nasopharynx of human as a commensal but causing pneumococcal diseases opportunistically, such as pneumonia, bacteremia, meningitis, and otitis media (Bogaert et al., 2004; Weiser et al., 2018). As an important amino acid, except for direct use for protein translation, methionine is the precursor of formyl methionine, the first building block for bacterial protein translation (Hondorp and Matthews, 2013) and *S*-adenosylmethionine (SAM), the methyl donor for the synthesis of DNA, RNA and proteins (Sperandio et al., 2007). Our previous studies show that the deletion of methionine synthesis gene *metE* plus limited methionine supply (1 μg/mL) in CDM causes total starvation in *S. pneumoniae* (Zhang et al., 2021, 2023). In this condition, methionine acquisition only comes from uptake by methionine transporter(s). When the limited methionine is exhausted, bacteria enter into methionine total starvation.

Although methionine is not an essential amino acid for pneumococcal growth, no methionine supply attenuates pneumococcal growth in CDM (Haertel et al., 2012). The work by Haertel et al. (2012) reveals that in the 20 amino acids, 8 amino acids are essential for pneumococcal growth in CDM. They are arginine, cysteine, histidine, glycine, glutamine, isoleucine, leucine and valine (Haertel et al., 2012). However, no supply of some of the remaining 12 amino acids (not essential) attenuates pneumococcal growth, especially for glutamate, proline or methionine (Haertel et al., 2012). Without exogenous supply, bacteria only rely on the precursors to synthesize the demanded amino acids. Similar phenomena were also found in Group B Streptococci. The absence of a certain amino acid in the media caused growth attenuation in some strains of Group B Streptococci (Milligan et al., 1978). Even for *Escherichia coli*, the removal of serine from medium reduced 22% of specific growth rate (Maser et al., 2020).

This intermediate growth state is characterized by no supply of a certain nutrient and synthesis of this nutrient by bacteria, and the growth yield is less than the full growth. We name this intermediate growth state semi-starvation. How bacteria adapt to semi-starvation is a fascinating question. In total starvation of amino acids, the alarmones guanosine 5′-monophosphate 3′-diphosphate (pGpp), guanosine tetraphosphate (ppGpp) and guanosine pentaphosphate (pppGpp), collectively referred to as (pp)pGpp (Cashel and Gallant, 1969; Haseltine and Block, 1973; Atkinson et al., 2011) are involved in DNA replication repression and rRNA synthesis inhibition (Potrykus and Cashel, 2008). In total starvation, bacteria try to survive, but not continue to grow. However, in semi-starvation, bacteria still try to grow. Our recent study shows that in methionine total starvation, pneumococcal cytoplasm is acidified and intracellularly accumulated glutamine balances intracellular pH to a mildly acidified level to enhance bacterial survival (Zhang et al., 2023). Glutamine can be deaminated to release ammonia that can neutralize protons (Lu et al., 2013). Whether intracellular pH homeostasis plays a role in pneumococcal growth under semi-starvation needs to be studied.

Intracellular pH is typically homeostatic in bacteria due to its important role in influencing enzyme activity, nucleic acid structure, redox potential, and secretion system activity (Veine et al., 1998; Slonczewski et al., 2009; Kenney, 2019). Unlike acidophiles and alkaliphiles that have an acidic and alkaline intracellular pH, respectively, (Matin et al., 1982; Sturr et al., 1994), neutralophiles usually keep their intracellular acidity closer to neutrality. For example, *E. coli* maintains the intracellular pH at 7.4–7.8 when it is cultured in pH 5–9 (Slonczewski et al., 2009). Intracellular pH homeostasis is important for bacterial growth (Slonczewski et al., 2009). However, at least two questions still remain to be answered. One is that to maintain a full growth, to what extent bacteria can withstand the fluctuation of intracellular pH. Another is that why this homeostasis is important for bacterial growth. In other words, unbalanced intracellular pH influence what to attenuate bacterial growth.

In this paper, we found that methionine semi-starvation did lead to intracellular acidification of *S. pneumoniae*. The optimal intracellular pH (~7.6) is extremely important for pneumococcal full-speed growth. Even a tiny change of intracellular pH caused growth attenuation. In total starvation, glutamine balances intracellular pH to a moderately acidified level (~7.2) to enhance bacterial survival but not growth (Zhang et al., 2023). In semi-starvation, glutamine elevates intracellular pH to enhance pneumococcal growth. With excessive glutamine, pneumococcal intracellular pH was increased to the optimal level (~7.6) and bacteria had a full growth even without methionine supply. We try to reveal how glutamine functions in methionine semi-starvation and how intracellular pH influences pneumococcal growth. Pneumococcal growth in blood is critical for the septicemia elicited by it (Yuste et al., 2005). In human blood, methionine is limited while glutamine is abundant (Bergstrom et al., 1974). *S. pneumoniae* may utilize this strategy of enhanced growth by glutamine in blood, which provides us new drug targets to treat pneumococcal disease.

## Results

### Intracellular acidification occurs under semi-starvation of methionine

Our previous work shows that deletion of methionine synthesis gene *metE* plus limited methionine supply (1 μg/mL) resulted in significant attenuation of pneumococcal growth (Zhang et al., 2023). In this culture, methionine acquisition only comes from exogenous supply. Metabolomics data show that after 6 h’ culture, intracellular methionine level of *metE* mutant is only 3% of that in Wild-type (WT) strain (Zhang et al., 2023), indicating the exhaustion of methionine. Besides this total starvation, bacteria could encounter another condition when methionine is not supplemented, while methionine synthesis functions well. In this condition, methionine acquisition only comes from synthesis.

**Figure 1:**
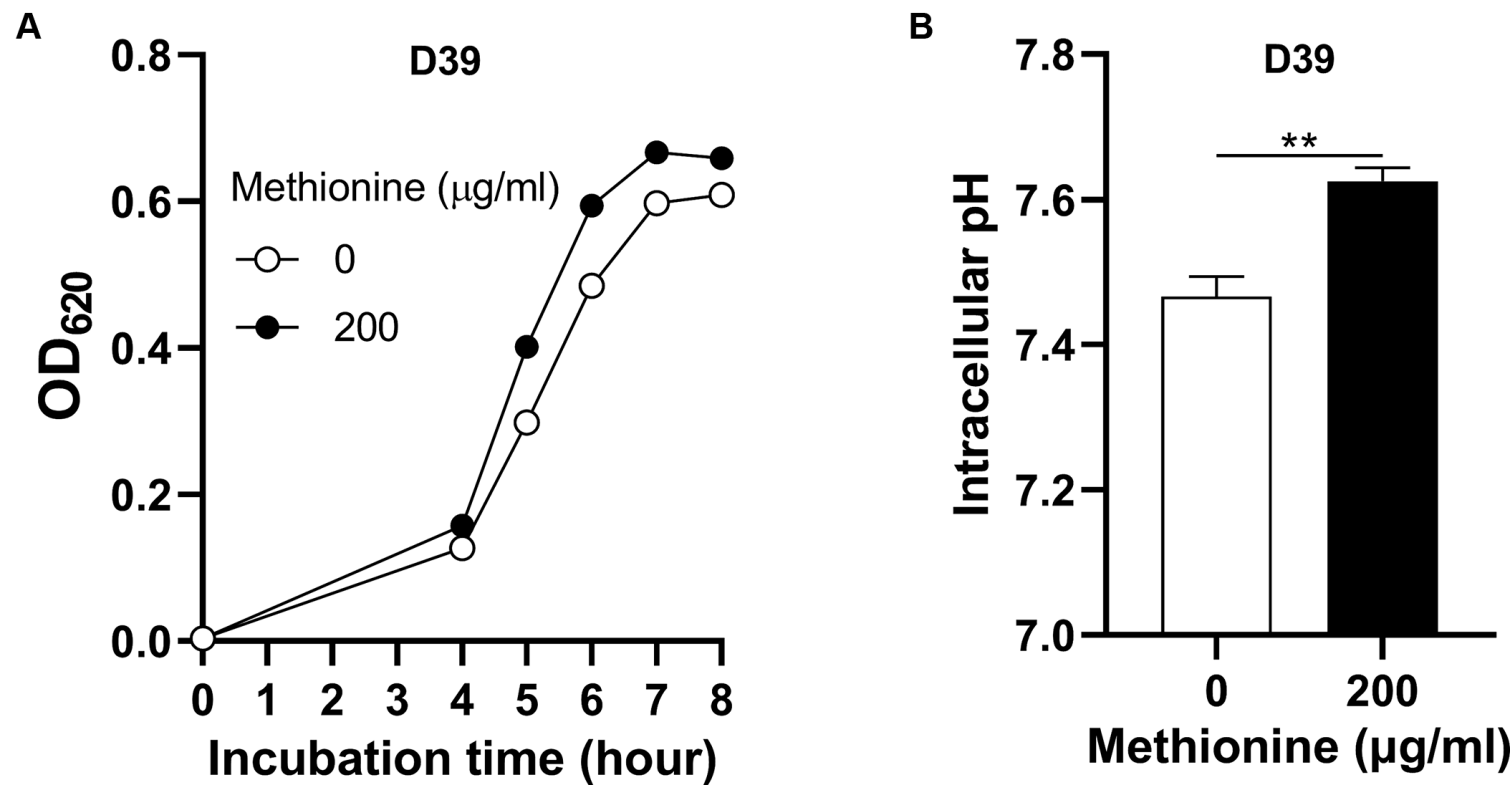
Intracellular acidification under methionine semi-starvation. **(A)** Growth attenuation of *S. pneumoniae* D39 under methionine semi-starvation. D39 growth in CDM with no or 200 μg/mL methionine was assessed by optical density at 620 nm (OD_620_) at various time points after inoculation. **(B)** Intracellular acidification of D39 under methionine semi-starvation. Intracellular pH of D39 cultured in CDM with no or 200 μg/mL methionine was determined at 6 h post inoculation. ** means *P* value < 0.01.

To determine how *S. pneumoniae* grows in this culture without methionine supply, *S. pneumoniae* D39 WT strain (D39) was cultured in CDM with no methionine or 200 μg/mL methionine (standard concentration). Bacterial growth with no methionine was attenuated significantly than growth with sufficient methionine (Figure 1A). Each point of growth curves represents the mean OD_620_ value of three replicates at each time point. The error bars of the growth curves were too short to be exhibited (also for other growth data in this paper). After 6 h’ culture, the OD_620_ values of bacteria cultured with no or 200 μg/mL methionine were 0.485 and 0.595, respectively. We name this weakened growth semi-starvation. Previously, we found that methionine total starvation induces intracellular acidification (Zhang et al., 2023). However, it is still unknown whether it happens under semi-starvation. Intracellular pH of bacteria cultured with no or 200 μg/mL methionine was 7.47 and 7.62, respectively, at 6 h post inoculation (Figure 1B). Obviously, intracellular acidification happened under methionine semi-starvation. Glutamine was previously found to be extremely important for pH balance under total starvation, in which massive intracellular glutamine accumulation was observed. Despite lower level, glutamine accumulation was also observed under semi-starvation (Zhang et al., 2023), indicating the function of glutamine for pneumococcal growth under semi-starvation. We next examined whether glutamine is important for pneumococcal growth under semi-starvation.

### Glutamine enhances pneumococcal growth and elevates intracellular pH under methionine semi-starvation

**Figure 2:**
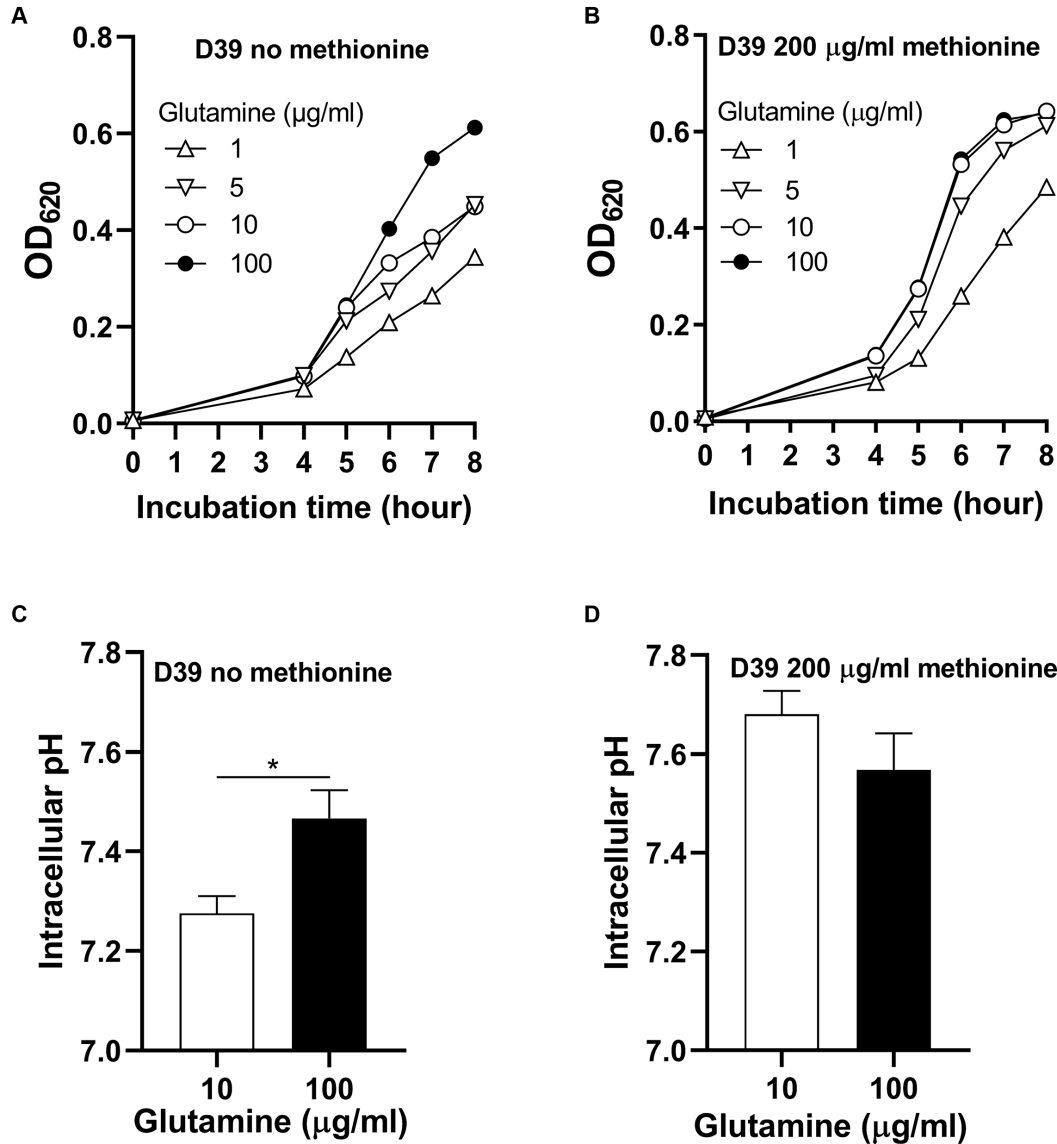
Growth enhancement and intracellular pH elevation by glutamine under methionine semi-starvation. **(A)** Growth enhancement of D39 under methionine semi-starvation by glutamine supply. D39 growth in CDM with no methionine and 1, 5, 10, or 100 μg/mL glutamine was determined by OD_620_ value at various time points. **(B)** Growth of D39 with sufficient methionine and different concentrations of glutamine. D39 growth in CDM with 200 μg/mL methionine and 1, 5, 10, or 100 μg/mL glutamine was determined by OD_620_ value at various time points. **(C)** Intracellular pH elevation by glutamine under methionine semi-starvation. Intracellular pH of D39 cultured with no methionine and 10 or 100 μg/mL glutamine was determined at 6 h post inoculation. **(D)** Intracellular pH of D39 cultured with sufficient methionine and different concentrations of glutamine. Intracellular pH of D39 cultured with 200 μg/mL methionine and 10 or 100 μg/mL glutamine was determined at 6 h post inoculation. *means *p* value < 0.05.

To know whether glutamine is important for pneumococcal growth under methionine semi-starvation, we firstly determined bacterial demand for glutamine in the culture with no methionine or sufficient methionine (200 μg/mL). The standard concentration of glutamine in CDM is 100 μg/mL. With no methionine, D39 was cultured with different concentrations of glutamine. After 8 h’ culture, the OD_620_ values of bacteria cultured with 1, 5, and 10 μg/mL glutamine were 0.344, 0.453 and 0.449 respectively, which are 56.2, 73.8, and 73.2% of the culture with 100 μg/mL glutamine (0.613) (Figure 2A). With 200 μg/mL methionine, the OD_620_ values of bacteria cultured with 1, 5, or 10 μg/mL glutamine were 0.485, 0.613, and 0.642, respectively, at 8 h post inoculation, which are 75.9, 95.8, and 100.5% of the culture with 100 μg/mL glutamine (0.639) (Figure 2B). These data show that with sufficient methionine, bacterial demand for glutamine decreased. With sufficient methionine, 10 μg/mL glutamine was enough for pneumococcal full growth. Further decrease of glutamine concentration in CDM with 200 μg/mL methionine also caused less attenuation of growth than culture with no methionine. Based on these data, we noticed that more glutamine is demanded for pneumococcal growth when methionine is not supplied, which means a growth enhancement function of glutamine under methionine semi-starvation.

How glutamine enhances pneumococcal growth under methionine semi-starvation is the key question we need to answer in this work. Glutamine is able to increase intracellular pH, we therefore gave our key hypothesis that intracellular acidification caused by semi-starvation impaired bacterial growth and glutamine elevate intracellular pH to enhance bacterial growth. To verify this hypothesis, we firstly determined whether glutamine can elevate intracellular pH under methionine semi-starvation. With no methionine supply, intracellular pH of D39 cultured in CDM with 10 or 100 μg/mL glutamine was 7.28 and 7.47, respectively, (Figure 2C). Decreased supply of glutamine did decrease intracellular pH significantly under methionine semi-starvation. However, with 200 μg/mL methionine, there was no significant difference of intracellular pH between the culture with 10 and 100 μg/mL glutamine (Figure 2D). Obviously, glutamine specifically increases intracellular pH under semi-starvation. If glutamine increases intracellular pH to enhance growth, then intracellular pH homeostasis must be essential for pneumococcal full growth. We next determined the importance of this homeostasis for bacterial growth.

### Intracellular pH 7.6 is essential for full-speed growth of *Streptococcus pneumoniae*

**Figure 3:**
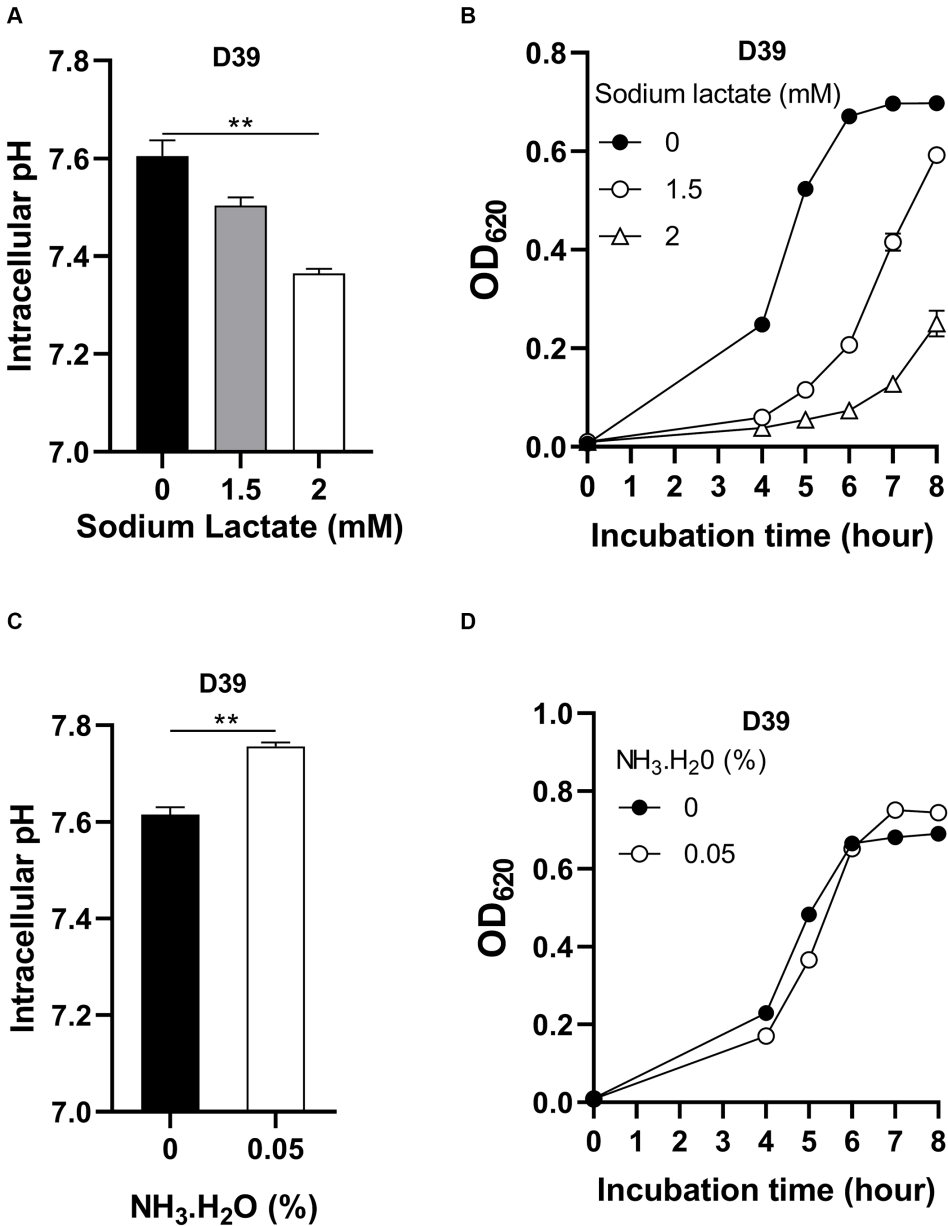
Impact of intracellular pH on pneumococcal growth. **(A)** Intracellular acidification by sodium lactate supply. Intracellular pH of D39 cultured in CDM with full nutrients and supplied with 0-, 1.5-, or 2-mM sodium lactate was determined at 6 h post inoculation. **(B)** Growth of D39 under intracellular acidification. Growth of D39 in CDM with supply of 0-, 1.5-, or 2-mM sodium lactate was determined by OD_620_ value at various time points. **(C)** Intracellular pH elevation by NH_3_.H_2_O. Intracellular pH of D39 cultured in CDM with full nutrients and supplied with no or 0.05% NH_3_.H_2_O was determined at 6 h post inoculation. **(D)** Growth of D39 under intracellular basification. Growth of D39 in CDM with supply of no or 0.05% NH_3_.H_2_O was determined by OD_620_ value at various time points. ** means *P* value < 0.01.

Although intracellular pH homeostasis was important for bacterial growth (Slonczewski et al., 2009), which range of this homeostasis has not been investigated. To determine if there exists an optimal intracellular pH for pneumococcal growth, D39 were cultured in CDM with full nutrients and supplemented with different concentrations of sodium lactate. Lactate is a weak organic acid, which can release H^+^ in the cytoplasm to cause intracellular acidification (Russell and DiezGonzalez, 1998). With no sodium lactate, the intracellular pH of D39 is 7.60 (Figure 3A). Supplement of 1.5 mM and 2 mM sodium lactate led to the decrease of intracellular pH to 7.50 and 7.36, respectively, (Figure 3A). This intracellular acidification impaired bacterial growth significantly. After 6 h’ culture with 0-, 1.5-, or 2-mM sodium lactate, the OD_620_ values of bacteria were 0.671, 0.207, and 0.073, respectively, (Figure 3B). Even a 0.1 drop of intracellular pH caused severe growth attenuation.

We also determined the impact of intracellular pH increment on bacterial growth by adding NH_3_.H_2_O. Supplement of 0.05% NH_3_.H_2_O increased intracellular pH from 7.62 to 7.76 (Figure 3C). This increase of intracellular pH also attenuated pneumococcal growth. After 5 h’ culture with no or 0.05% NH_3_.H_2_O, the OD_620_ values of bacteria were 0.483 and 0.366, respectively, (Figure 3D). Interestingly, this increase of intracellular pH caused less growth attenuation than growth with decreased intracellular pH. At 8 h post inoculation, the OD_620_ value of bacterial culture with supply of NH_3_.H_2_O was even higher than without NH_3_.H_2_O supply and there was no significant difference between their intracellular pH (Supplementary Figure S1). After 6 h’ cultivation, the nitrogen source might become limited and NH_3_ might be utilized by bacteria as a nitrogen source for growth. Therefore, intracellular pH was not elevated and bacteria grew better by NH_3_.H_2_O supply at 6 to 8 h post inoculation.

Taken together, these data show that even tiny disturbance of intracellular pH impairs bacterial growth, which emphasizes the importance of intracellular pH homeostasis for pneumococcal full growth. We can also conclude that intracellular pH ~ 7.6 is essential for pneumococcal full growth. In other words, this homeostasis range is very narrow. Currently, we know that semi-starvation induces intracellular acidification that impairs pneumococcal growth and glutamine elevates intracellular pH to enhance bacterial growth. The further question is how glutamine works to elevate intracellular pH. Glutamine can be deaminated to release NH_3_, which can neutralize proton (Lu et al., 2013). Therefore, to find the glutamine deaminase is the key to solve this question.

### Glutamine deamination elevates intracellular pH under methionine semi-starvation to enhance pneumococcal growth

**Figure 4:**
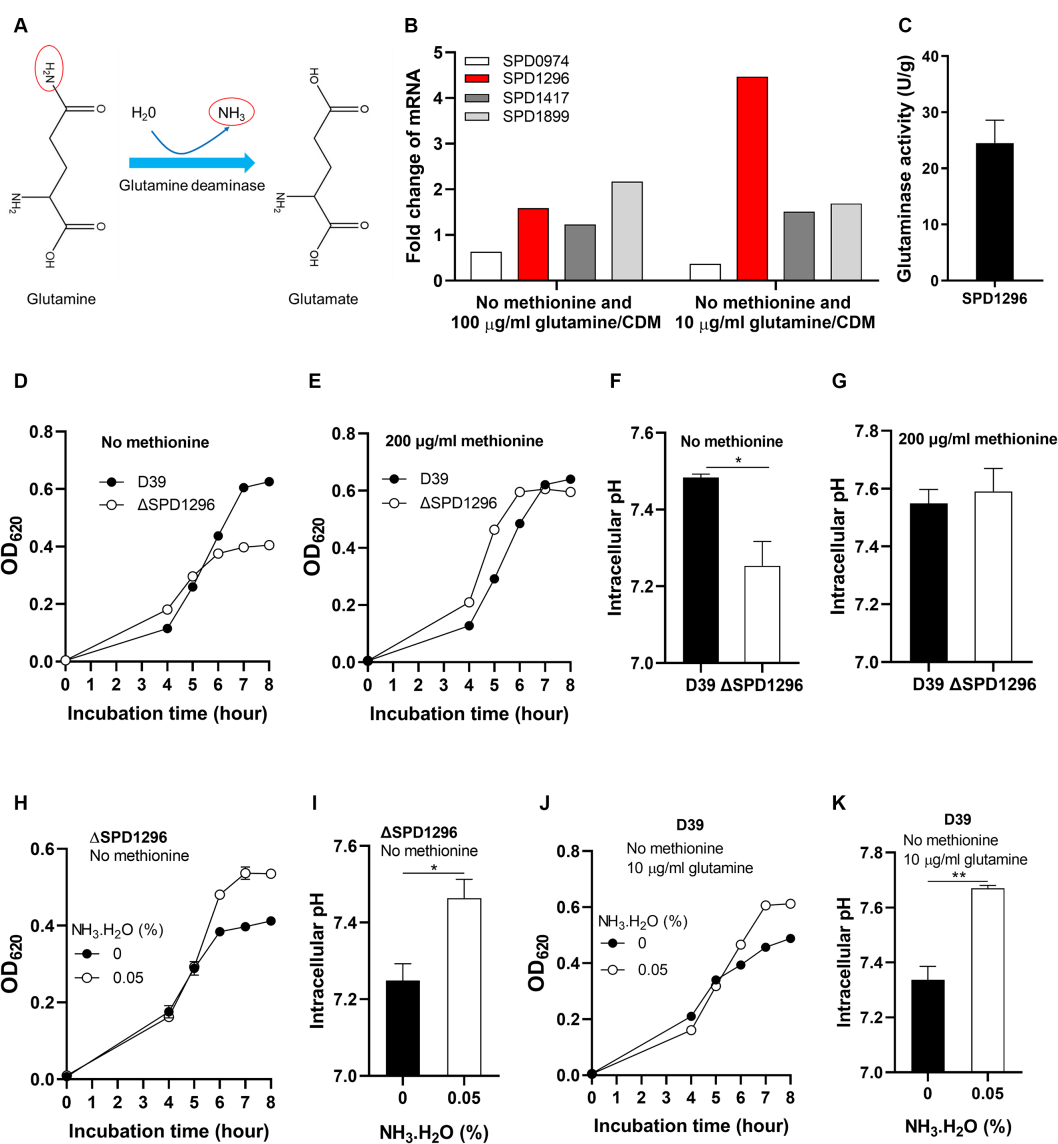
Enhanced growth under methionine semi-starvation by glutamine deamination. **(A)** Diagram of glutamine deamination. In the presence of glutamine deaminase and H_2_O, the NH_2_- at the C-terminus of glutamine is released to form NH_3_ and glutamine is converted to glutamate. **(B)** Transcription change of glutamine deaminase genes under methionine semi-starvation. mRNA levels of SPD0974, SPD1296, SPD1417, and SPD1899 were compared between the culture of D39 in CDM with no methionine and with full nutrients or no methionine plus 10 μg/mL glutamine and full nutrients. **(C)** Glutaminase activity (U/g protein) of SPD1296. **(D)** Importance of SPD1296 for pneumococcal growth under methionine semi-starvation. Growth of D39 and SPD1296 deletion mutant (ΔSPD1296) in CDM with no methionine was determined by OD_620_ value at various time points. **(E)** Dispensable role of SPD1296 for pneumococcal growth in full nutrients. Growth of D39 and SPD1296 deletion mutant in CDM with full nutrients was determined by OD_620_ value at various time points. **(F)** Intracellular acidification of SPD1296 mutant under methionine semi-starvation. Intracellular pH of D39 and SPD1296 mutant cultured in CDM with no methionine was determined at 6 h post inoculation. **(G)** Intracellular pH of D39 and SPD1296 mutant cultured in CDM with full nutrients. Intracellular pH was determined at 6 h post inoculation. **(H)** Enhanced growth of SPD1296 mutant under methionine semi-starvation by NH_3_.H_2_O supplement. Growth of SPD1296 mutant in CDM with no methionine and supplied with no or 0.05% NH_3_.H_2_O was determined by OD_620_ value at various time points. **(I)** Intracellular pH elevation of SPD1296 mutant under methionine semi-starvation by NH_3_ supplement. Intracellular pH of SPD1296 mutant cultured in CDM with no or 0.05% NH_3_.H_2_O was determined at 6 h post inoculation. **(J)** Enhanced growth of D39 under methionine semi-starvation by NH_3_.H_2_O supplement. Growth of D39 in CDM with no methionine and 10 μg/mL glutamine and supplied with no or 0.05% NH_3_.H_2_O was determined by OD_620_ value at various time points. **(K)** Intracellular pH elevation of D39 under methionine semi-starvation by NH_3_.H_2_O supplement. Intracellular pH of D39 cultured in M0Q10 with no or 0.05% NH_3_.H_2_O was determined at 6 h post inoculation. *means *p* value < 0.05. ** means *p* value < 0.01.

The deamination of glutamine is illustrated in Figure 4A. With one molecule H_2_O, the terminal NH_2_- of glutamine is replaced by -OH and NH_3_ is released. This reaction catalyzed by glutamine deaminase converts glutamine to glutamate. Four putative glutamine deaminases were identified. They are SPD0974, SPD1296, SPD1417, and SPD1899 (Zhang et al., 2023). To determine which deaminase contributes to the deamination of glutamine under methionine semi-starvation, the transcription levels of the four genes were compared in different cultures, no methionine and 10 μg/mL glutamine (M0Q10), no methionine and 100 μg/mL glutamine (M0Q100), and 200 μg/mL methionine and 100 μg/mL glutamine (CDM). Compared to CDM, transcription of SPD1296 and SPD1899 in M0Q100 was up-regulated (>1.5-fold), 1.6- and 2.2-fold, respectively, (Figure 4B). Each bar for qRT-PCR data represents the mean value of three technically repeated samples and each qRT-PCR experiment was repeated once (same for other qRT-PCR data in this paper). Further decrease of glutamine concentration (M0Q10) up-regulates the transcription of SPD1296 further (4.5-fold of that in CDM, Figure 4B). However, transcription of other 3 genes was not up-regulated further. These data indicate the important role of SPD1296 for pneumococcal growth under methionine semi-starvation.

The function of SPD1296 as glutamine deaminase was verified by *in vitro* deamination reaction assay. Purified SPD1296 with N-terminal 6 × His tag was shown in the sodium dodecyl sulfate (SDS)-polyacrylamide gel stained by Coomassie blue (Supplementary Figure S2). The reaction system contained glutamine, buffer and crude enzyme. The product (glutamate) was detected by HPLC–MS. Successful detection of glutamate from the reaction showed the glutamine deaminase activity of SPD1296. The specific activity of SPD1296 as a glutamine deaminase was 24.5 U/g (Figure 4C). By comparing the growth of D39 and SPD1296 deletion mutant (ΔSPD1296) with no or 200 μg/mL methionine, we observed that deletion of SPD1296 impaired bacterial growth severely when no methionine was supplied (Figure 4D). However, with sufficient methionine, SPD1296 mutant grew even better than D39 (Figure 4E). These growth data confirmed the importance of SPD1296 for pneumococcal growth under methionine semi-starvation. Deletion of SPD1296 decreased intracellular pH from 7.48 to 7.25 under semi-starvation (Figure 4F). However, it did not lead to intracellular acidification when methionine was sufficient (Figure 4G). These results show that SPD1296, as a glutamine deaminase, contributes to bacterial growth under methionine semi-starvation by increasing intracellular pH.

To confirm the function of SPD1296 as glutamine deaminase to increase intracellular pH, NH_3_.H_2_O was added into the culture of SPD1296 mutant. Supply of 0.05% NH_3_.H_2_O enhanced the growth of SPD1296 mutant without methionine. After 8 h’ culture, the OD_620_ value of SPD1296 mutant cultured with no or 0.05% NH_3_.H_2_O was 0.412 and 0.535, respectively, (Figure 4H). Supply of NH_3_.H_2_O increased intracellular pH of SPD1296 mutant from 7.25 to 7.46 (Figure 4I). These data indicated that deletion of SPD1296 reduced intracellular NH_3_ level and supply of NH_3_.H_2_O replenished the diminished NH_3_ to increase intracellular pH. Similarly, supply of NH_3_.H_2_O restored the growth of D39 cultured with no methionine and 10 μg/mL glutamine (Figure 4J). This supply of NH_3_ increased intracellular pH from 7.34 to 7.67 (Figure 4K).

By far, these results illustrate that glutamine increases intracellular pH by deamination to enhance pneumococcal growth under semi-starvation. However, we raised another question that why 100 μg/mL glutamine (standard concentration in CDM) did not rescue bacterial growth with no methionine to the level of growth with full nutrients. We speculated that 100 μg/mL glutamine is not enough to fully rescue bacterial growth. In other words, with sufficient glutamine, semi-starvation would not occur. We next tried to verify this.

### Insufficient glutamine supply results in methionine semi-starvation

**Figure 5:**
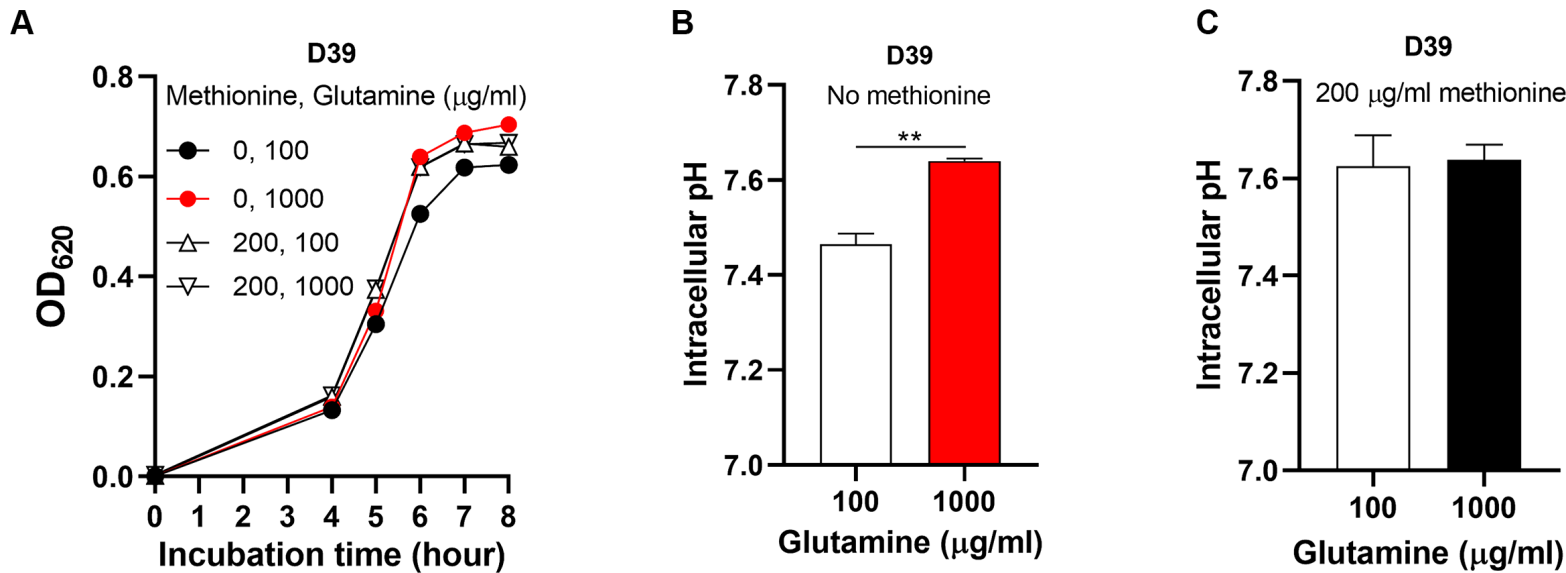
Abolished semi-starvation by excessive glutamine supply. **(A)** Recovered growth of D39 under methionine semi-starvation by excessive glutamine supply. Growth of D39 cultured in CDM with no methionine and 100 μg/mL glutamine, no methionine and 1,000 μg/mL glutamine, 200 μg/mL methionine and 100 μg/mL glutamine or 200 μg/mL methionine and 1,000 μg/mL glutamine was determined by OD_620_ value at various time points. **(B)** Restored intracellular pH under methionine semi-starvation by excessive glutamine supply. Intracellular pH of D39 cultured in CDM with no methionine and 100 μg/mL glutamine or no methionine and 1,000 μg/mL glutamine was determined at 6 h post inoculation. **(C)** Intracellular pH of D39 cultured in CDM with 200 μg/mL methionine and 100 μg/mL glutamine or 200 μg/mL methionine and 1,000 μg/mL glutamine. Intracellular pH was determined at 6 h post inoculation. ** means *P* value < 0.01.

The intracellular pH of D39 cultured with no methionine and 100 μg/mL glutamine is still lower than D39 cultured with full nutrients. Supply of more glutamine (over 100 μg/mL) may further enhance bacterial growth and elevate its intracellular pH. To verify this, we firstly compared the growth of D39 with no methionine and 100 μg/mL glutamine (M0Q100), no methionine and 1,000 μg/mL glutamine (M0Q1000), 200 μg/mL methionine and 100 μg/mL glutamine (M200Q100), and 200 μg/mL methionine and 1,000 μg/mL glutamine (M200Q1000). After 6 h’ culture, the OD_620_ values of M0Q100, M0Q1000, and M200Q100 were 0.526, 0.640, and 0.620, respectively, (Figure 5A). Supply of more glutamine (M0Q1000) did further enhance the growth of D39 with no methionine (M0Q100) to the level of growth with full nutrients (M200Q100). With this excessive glutamine (1,000 μg/mL) supply, growth attenuation in methionine semi-starvation disappeared. Interestingly, with sufficient methionine (200 μg/mL), supply of more glutamine (M200Q1000) did not enhance its growth. After 6 h’ culture, the OD_620_ value of M200Q1000 was 0.619 (Figure 5A). These data show that 100 μg/mL glutamine is enough for pneumococcal growth with 200 μg/mL methionine, but not enough for growth with no methionine. Growth enhancement by glutamine only works when methionine is not supplied.

After 6 h’ culture, the intracellular pH of M0Q1000 was 7.64, significantly higher than M0Q100 (pH 7.46) (Figure 5B). This shows that insufficient glutamine supply caused the intracellular acidification in semi-starvation, thus attenuating bacterial growth. Interestingly, this increase of intracellular pH did not happen in the culture with sufficient methionine. The intracellular pH of M200Q100 and M200Q1000 was 7.62 and 7.64, respectively, (Figure 5C). This shows that although glutamine elevates intracellular pH, it will not elevate intracellular pH over the optimal level (~7.6). Taken together, these results show that it is the intracellular acidification that causes semi-starvation. Excessive glutamine (1,000 μg/mL) elevated intracellular pH to the optimal level to eliminate the growth attenuation under methionine semi-starvation. Currently, we know that glutamine elevates intracellular pH to enhance pneumococcal growth with no methionine. Obviously, intracellular acidification attenuates pneumococcal growth under methionine semi-starvation. The question is that which cellular process is influenced by cytoplasmic acidification.

### Glutamine elevates translation level to enhance pneumococcal growth under semi-starvation

**Figure 6:**
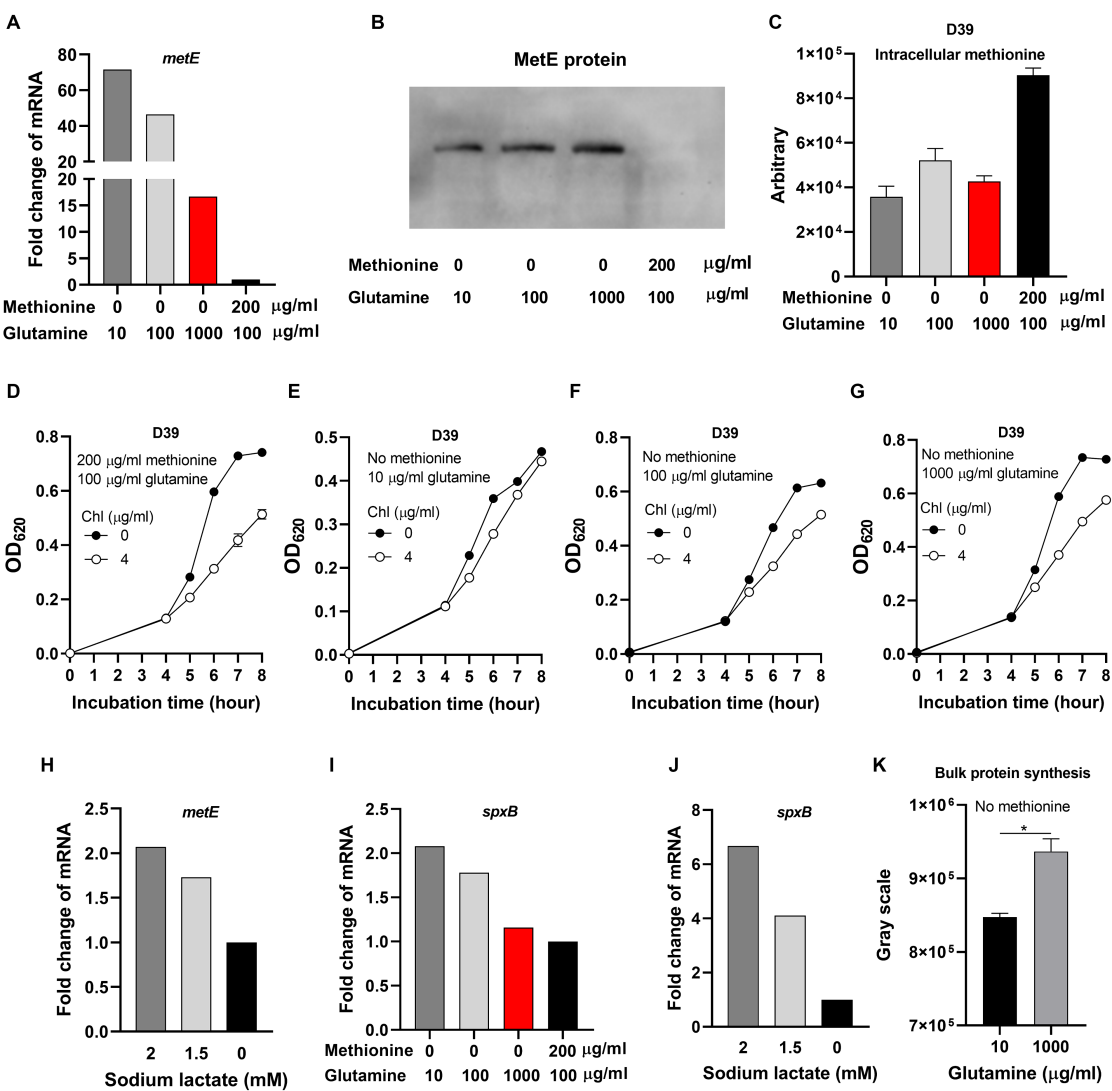
Inhibited translation under methionine semi-starvation. **(A)** Comparison of *metE* transcription level in D39 cultured with different concentrations of methionine and glutamine. mRNA levels of *metE* were compared among the cultures with no methionine and 10 μg/mL glutamine, no methionine and 100 μg/mL glutamine, no methionine and 1,000 μg/mL glutamine, and 200 μg/mL methionine and 100 μg/mL glutamine, respectively. mRNA of *metE* in 200 μg/mL methionine and 100 μg/mL glutamine was set as 1. **(B)** Comparison of MetE protein levels in the cultures in **(A)**. One OD bacteria were collected at 6 h post inoculation. MetE protein with 6 × His tag was detected by western blot. **(C)** Intracellular methionine amount of D39 cultured as in **(A)**. Intracellular methionine of bacteria cultured at 6 h post inoculation was quantified by Liquid Chromatography Tandem-mass Spectrometry (LC–MS/MS). **(D–G)** Reduced sensitivity to chloramphenicol under methionine semi-starvation. Growth of D39 cultured in 200 μg/mL methionine and 100 μg/mL glutamine with no or 4 μg/mL chloramphenicol (chl) **(D)**, no methionine and 10 μg/mL glutamine with no or 4 μg/mL chloramphenicol (chl) **(E)**, no methionine and 100 μg/mL glutamine with no or 4 μg/mL chloramphenicol (chl) **(F)**, and no methionine and 1,000 μg/mL glutamine with no or 4 μg/mL chloramphenicol (chl) **(G)** respectively was determined by OD_620_ value at various time points. **(H)** Comparison of *metE* transcription level in D39 cultured with full nutrients (CDM) and supply of different concentration of sodium lactate. mRNA levels of *metE* were compared among the cultures supplied with 0-, 1.5-, and 2-mM sodium lactate. mRNA of *metE* in 0 mM sodium lactate was set as 1. **(I)** Comparison of *spxB* transcription level in D39 cultured with different concentrations of methionine and glutamine. mRNA levels of *spxB* in D39 were compared among the cultures in **(A)**. mRNA of *spxB* in 200 μg/mL methionine and 100 μg/mL glutamine was set as 1. **(J)** Comparison of *spxB* transcription level in D39 cultured with full nutrients (CDM) and supply of different concentration of sodium lactate. mRNA levels of *spxB* were compared among the cultures with 0-, 1.5-, and 2-mM sodium lactate. mRNA of *spxB* in 0 mM sodium lactate was set as 1. **(K)** Enhanced translation by glutamine supply under methionine semi-starvation. At 6 h post inoculation, newly synthesized proteins in 2 optical density (OD) D39 cultured with no methionine and 10 μg/mL glutamine or no methionine and 1,000 μg/mL glutamine were detected by L-Azidohomoalanine (AHA) click labeling assay. The gray scales of total proteins detected were calculated by ImageJ_v1.8.0. *means *p* value < 0.05.

Although glutamine elevates intracellular pH to enhance bacterial growth, we still do not know which cellular process is rescued by glutamine. Under methionine semi-starvation, methionine acquisition only comes from synthesis. We hypothesized that intracellular acidification down-regulates the transcription of methionine synthesis gene *metE* and the growth attenuation is caused by insufficient methionine synthesis. To test this hypothesis, we compared the transcription of *metE* in the culture of M0Q10, M0Q100, M0Q1000, and M200Q100. The transcription of *metE* in M0Q1000 was 16.7-fold of *metE* in M200Q100 (Figure 6A). In our hypothesis, with the decreased concentrations of glutamine, the transcription level of *metE* decreased. Surprisingly, decreased supply of glutamine up-regulated the transcription of *metE*. The transcription of *metE* in M0Q100 and M0Q10 was 46.5-fold and 71.5-fold of that in M200Q100, respectively, (Figure 6A). To determine whether more mRNA of *metE* produces more MetE protein, MetE protein (with 6 × His tag) from 1 optical density (OD) bacteria cultured in M0Q10, MOQ100, M0Q1000, or M200Q100 was determined at 6 h post inoculation by western blot. MetE protein was not detected in bacteria cultured in M200Q100 (Figure 6B), which shows the dispensable role of MetE when sufficient methionine is supplied. When methionine was not supplied, with the decreasing concentration of glutamine (from 1,000 to 10 μg/mL), MetE protein level decreased. The gray scales of MetE protein in M0Q1000, M0Q100, and M0Q10 are 66,091, 58,849 and 41,933, respectively. More *metE* mRNA did not produce more MetE protein, but conversely, less MetE protein (Figure 6B). These data show that the translation of MetE was enhanced by glutamine supply in the culture with no methionine.

To determine whether intracellular methionine level was reduced due to the shortage of glutamine under methionine semi-starvation, intracellular methionine quantification was determined by Liquid Chromatography Tandem-mass Spectrometry (LC–MS/MS). Interestingly, there is no significant difference of intracellular methionine amount among the cultures of M0Q10, M0Q100, and M0Q1000 (Figure 6C). Besides, intracellular methionine levels in these cultures were significantly lower than that in M200Q100 (Figure 6C). These results provide us with several important information. Firstly, methionine synthesis ability is limited. Even with excessive glutamine and optimal intracellular pH (~7.6), intracellular methionine level of M0Q1000 could not reach (still lower than) the level of M200Q100. Secondly, since the growth of M0Q1000 was as well as M200Q100, less intracellular methionine in M0Q1000 does not mean worse growth. It is not the less intracellular methionine but intracellular acidification that causes growth attenuation in methionine semi-starvation. Thirdly, supplement of glutamine elevates intracellular pH but did not enhance methionine synthesis ability under methionine semi-starvation.

Based on the above data, it seems that translation inhibition contributes to growth attenuation under methionine semi-starvation. Chloramphenicol inhibits the translation of bacteria. If the translation of *S. pneumoniae* was inhibited, then the inhibition function of chloramphenicol (chl) would be weakened. After incubation for 6, 7, and 8 h, the OD_620_ values of M200Q100 with no chloramphenicol were 0.596, 0.729 and 0.741, respectively, (Figure 6D). At the same time, the OD_620_ values of M200Q100 with 4 μg/mL chloramphenicol were 0.313, 0.418, and 0.514, respectively. The percentages of OD_620_ values (chl/no chl) were 52.5, 57.3, and 69.3%, respectively, at 6, 7, and 8 h post inoculation, respectively. The growth of D39 in M200Q100 was weakened significantly by chloramphenicol addition (Figure 6D). However, the growth of D39 in M0Q10 was not weakened by addition of chloramphenicol (Figure 6E). After incubation for 6, 7, or 8 h, the percentages of OD_620_ values (chl/no chl) at 6, 7, and 8 h were 77.4, 92.3, and 95.1%, respectively. However, for M0Q100, the percentages of OD_620_ values (chl/no chl) at 6, 7, and 8 h were 69.5, 72.1, and 81.6%, respectively, (Figure 6F). For M0Q1000, the percentages of OD_620_ values (chl/no chl) at 6, 7, and 8 h were 62.9, 67.4, and 79.2%, respectively, (Figure 6G). These data show that with the increasing supply of glutamine in the culture of no methionine, the growth inhibition effect by chloramphenicol increased, which strongly suggest that glutamine supply increased translation level under methionine semi-starvation.

Whether intracellular acidification under methionine semi-starvation caused translation inhibition needs to be verified. Sodium lactate addition decreases intracellular pH (Figure 3A). Supplement of 1.5- or 2-mM sodium lactate in CDM increased the transcription of *metE* by 0.7- and 1.1-fold, respectively, (Figure 6H). We also tested another gene *spxB* that encodes a pyruvate oxidase catalyzing pyruvate, phosphate and O_2_ to acetyl phosphate, CO_2_ and H_2_O_2_ (Lisher et al., 2017). The transcription of *spxB* in M0Q1000, M0Q100, and M0Q10 was 1.2-, 1.8-, and 2.1-fold of that in M200Q100, respectively, (Figure 6I). Supply of 1.5- or 2-mM sodium lactate increased the transcription of *spxB* by 3.1-fold and 5.7-fold, respectively, (Figure 6J). These results indicate the attenuated translation by intracellular acidification. Glutamine may elevate intracellular pH to enhance translation.

In order to determine the impact of intracellular acidification on translation activity, we used L-Azidohomoalanine (AHA) click labeling assay to determine the newly synthesized global proteins. The mechanism of this method is the click reaction of azide and alkyne. AHA containing an azide moiety is a methionine analog that can be used as substrate for protein synthesis (incorporation). After incorporation of AHA into the newly synthesized proteins, biotin-alkyne was added. AHA and biotin-alkyne was connected by azide-alkyne cycloaddition. Then AHA could be detected by western blot using HRP-conjugated streptavidin that detects biotin. D39 was cultured in CDM with no methionine and 10 or 1,000 μg/mL glutamine. At 6 h post inoculation, the same amount of bacteria (2 OD) were collected for AHA click labeling assay. Most of the newly synthesized proteins (Supplementary Figure S3) are more abundant in bacteria cultured with no methionine and excessive (1,000 μg/mL) glutamine, compared with bacteria cultured with no methionine and 10 μg/mL glutamine. The bulk protein synthesis (shown by gray scale detected by western blot) of bacteria cultured with no methionine and 1,000 μg/mL glutamine is significantly more abundant than that of bacteria cultured with no methionine and 10 μg/mL glutamine (Figure 6K). These data show the increased translation activity by excessive glutamine supply. Since bacteria cultured with no methionine and 1,000 μg/mL glutamine has a higher intracellular pH, it suggests that intracellular acidification attenuates bacterial translation.

Low intracellular pH may decrease DNA replication. In order to determine the impact of intracellular acidification on replication activity, we used BeyoClick™ EdU (5-ethynyl-2′-deoxyuridine) cell proliferation kit (Beyotime Biotechnology, China) to determine the newly synthesized DNA. This method used the click reaction of azide labeled by a fluorescent probe and alkyne moiety contained in Edu. Edu is an analog of thymidine, which can be incorporated in to the newly synthesized DNA. D39 were cultured in CDM with no methionine and 10 or 1,000 μg/mL glutamine. At 6 h post inoculation, 1 OD bacteria were collected for Edu assay. Interestingly, the fluorescence was stronger in bacteria cultured with 10 μg/mL glutamine, compared with 1,000 μg/mL glutamine (Supplementary Figure S4). This indicates that low intracellular pH increases DNA replication. We speculated that this enhanced replication is also a way for compensating attenuated translation.

Taken together, we raised a model. In the culture of no methionine, bacterial cytoplasm acidified. This intracellular acidification down-regulates translation efficiency. To compensate for impaired translation, bacteria up-regulates the replication activity and the transcription of some genes (producing more mRNA). More mRNA means more protein. However, even more mRNA produces still less protein, which means the failure of this rescue. More glutamine supply increases intracellular pH to increase translation efficiency, thus diminishing the need for mRNA. Finally, this enhanced translation by glutamine supply rescued bacterial growth under methionine semi-starvation.

## Discussion

There are plenty of studies about nutrient starvation in bacteria. Most of these studies are about total starvation. For example, bacteria enter into stationary phase after exhausting nutrients in exponential phase (Llorens et al., 2010). Increased level of alternative sigma factor RpoS during stationary phase regulates the transcription of up to 10% genes to enhance the adaptation of *E. coli* to stresses (Weber et al., 2005). The stringent response is induced under amino acid starvation. The accumulated ppGpp down-regulates DNA replication and rRNA synthesis and promotes amino acid synthesis (Magnusson et al., 2005). However, the intermediate state between the fast-growing state and starvation state was ignored. In this work, the growth of *S. pneumoniae* in CDM without methionine supply was attenuated. Under this condition, bacteria still had an exponential growth phase but its growth yield was less than full growth with all nutrients supplied. We refer to this intermediate state between ceased growth and fast growth as semi-starvation. In semi-starvation, nutrient acquisition only comes from synthesis.

We hold the view that as long as the approach for getting the nutrient exists, bacteria will try to utilize this approach to increase the scale of their population. A big population may be extremely important for avoiding the clearance of this population under various environmental stresses. In total starvation, bacteria almost cease growth and pay their attention to how to survive for a longer time as a population. However, in semi-starvation, bacteria still try to grow. In methionine semi-starvation of *S. pneumoniae*, methionine synthesis still works. Synthesis of methionine needs cysteine as the precursor (Ferla and Patrick, 2014). However, without methionine supply, even with 413 μg/mL (the standard concentration in CDM) cysteine, bacteria grew worse than the full growth. The amount of intracellular methionine of bacteria in M0Q100 is 57.7% of that in M200Q100. It seems that insufficient methionine synthesis contributes to growth attenuation. Surprisingly, it is intracellular acidification but not insufficient methionine synthesis that causes growth attenuation. Previously, we found that methionine starvation causes intracellular acidification. In methionine semi-starvation, intracellular acidification also occurs, which indicates that as long as intracellular methionine amount is lower than methionine level in the culture supplied with sufficient methionine, bacterial cytoplasm will be acidified. Glutamine promotes bacterial growth by elevating intracellular pH. Excessive glutamine supply fully restored bacterial growth under methionine semi-starvation but did not increase intracellular methionine level. Therefore, it needs to be emphasized that this lower level of intracellular methionine is still able to support bacterial full growth. The prerequisite is that bacterial cytoplasm is not acidified.

Although glutamine functions by deamination to increase intracellular pH, the extents of this intracellular pH adjustment are quite different in total starvation and semi-starvation. Cytoplasmic acidification is more severe under methionine total starvation than under methionine semi-starvation. Glutamine prevents the over-acidification of cytoplasm and intracellular pH maintains at a moderately acidified level under methionine total starvation. Maintaining a moderately acidified cytoplasm may reduce the production of harmful compounds that contributes to bacterial death, thus enhancing bacterial survival. Therefore, the optimal intracellular pH for pneumococcal survival under total starvation is below 7.6. By suppling sodium lactate to decrease intracellular pH and NH_3_.H_2_O to increase intracellular pH, we observed that the optimal intracellular pH for pneumococcal growth is stringently confined to ~7.6. Even a tiny change of intracellular pH (0.1) significantly impaired bacterial growth. Under methionine semi-starvation, glutamine tries to elevate intracellular pH to ~7.6 to meet the demand for full-speed growth. The reason why semi-starvation occurs is that glutamine is not supplied sufficiently. The standard concentration of glutamine in CDM is 100 μg/mL. Normally, this concentration is high enough to support bacterial full-speed growth. Actually, when methionine is sufficient (200 μg/mL), only 10 μg/mL glutamine is enough for pneumococcal full-speed growth. However, when methionine was removed, 100 μg/mL glutamine was not enough to support full-speed growth of bacteria. In methionine semi-starvation, 100 μg/mL glutamine cannot elevate intracellular pH to 7.6. By adding excessive glutamine (1,000 μg/mL), intracellular pH increased to 7.6 and growth attenuation disappeared. Interestingly, when methionine was sufficient, 1,000 μg/mL glutamine in CDM did not further elevate intracellular pH and intracellular pH still remained at 7.6. The extra glutamine seems not to be utilized in this condition. This again emphasized the importance of the optimal intracellular pH (7.6) for pneumococcal full growth.

Our previous work has demonstrated that glutamine cannot be converted to methionine by isotope tracing (Zhang et al., 2023). In this paper, glutamine supply did not increase intracellular methionine level. The way of suppling excessive glutamine to recover bacterial growth under methionine semi-starvation is an interesting phenomenon. Actually, the increased population size means the exhaustion of methionine precursor cysteine. Is this an artificial phenomenon, or it can happen in the nature? In natural environment, the increasing concentration of glutamine may mean the possible source of other nutrients. In this condition, bacteria need to prepare to utilize the upcoming nutrients. Nutrients acquisition time may be very short. Only the immediate response to the nutrients makes bacteria grow as soon as possible. In semi-starvation, intracellular acidification prevents the immediate response of bacteria to nutrients. Excessive glutamine increases intracellular pH to the optimal level for growth and makes bacteria utilize the upcoming nutrients immediately and efficiently.

How intracellular pH influences bacterial growth is not well studied. The work by Zilberstein et al. (1984) shows that in a mutant of *E. coli* that is defective in pH homeostasis, the protein synthesis rate decreased to 50% after the shift of extracellular pH from 7.2 to 8.8 for 100 min and the DNA synthesis rate decreased to 60% after 3–5 min (Zilberstein et al., 1984). These results indicate the relationship between intracellular pH and translation or replication. In this study, the data suggested that pneumococcal overall transcription level increased and the overall translation rate decreased under methionine semi-starvation. These indicate that maintaining protein synthesis rate is an important function of intracellular pH homeostasis. In other words, protein synthesis is sensitive to cytoplasmic acidity. Disturbance of the homeostasis of intracellular pH decreases protein translation rate, thus attenuating bacterial growth. The up-regulation of transcription may be a try to enhance the production of protein by providing more mRNA. However, this try failed. In addition, the massive production of mRNA may consume too much energy, thus causing burden to bacterial growth.

**Figure 7:**
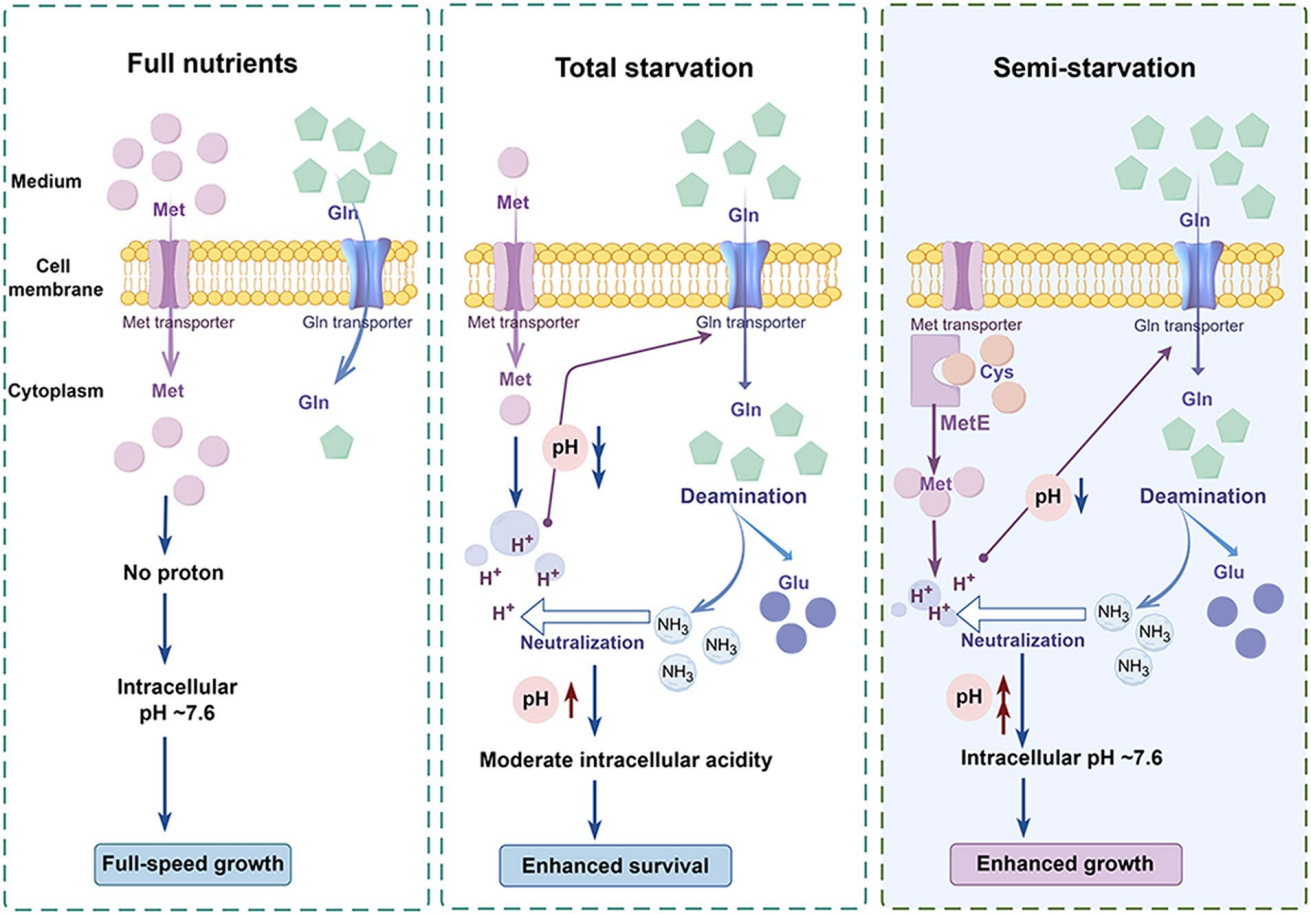
Model of enhanced bacterial survival under methionine total starvation and enhanced bacterial growth under methionine semi-starvation by glutamine. (By Figdraw.). When methionine (met) supply is sufficient, intracellular methionine level is high and bacterial cytoplasm is not acidified. Therefore, bacteria grow with full speed. When methionine is extremely limited and methionine synthesis does not work, intracellular methionine level becomes very low after a short time of culture. In this total starvation condition, bacterial cytoplasm is acidified severely and bacteria almost cease growth. This intracellular acidification induces the uptake of glutamine (gln), which elevates intracellular pH by deamination to a moderate acidity level that is most beneficial for bacterial survival. When no methionine is supplied and methionine synthase MetE works, intracellular methionine level is still lower than the full nutrients condition. Under this semi-starvation, the acidification of cytoplasm also occurs, which attenuates bacterial growth. With excessive glutamine supply, intracellular pH is elevated to ~7.6, which recovers bacterial growth fully.

The functions of glutamine for enhanced pneumococcal survival under total starvation and enhanced pneumococcal growth under methionine semi-starvation were summarized in Figure 7. When methionine is sufficient, bacteria acquire methionine by methionine transporter. In this condition, intracellular methionine level is high and intracellular acidification will not be induced. Therefore, intracellular pH maintains at ~7.6, the optimal value for growth. This normal intracellular pH will not up-regulate the transport of glutamine. Therefore, intracellular glutamine level is not high. When methionine is limited and meanwhile methionine synthesis cannot work, methionine acquisition comes from uptake. However, when the limited methionine is depleted, bacteria enter into total starvation. In total starvation, intracellular proton level increases, leading to severe drop of intracellular pH. This cytoplasmic acidification causes massive glutamine uptake. The accumulated glutamine is deaminated to release ammonia that neutralize protons to increase intracellular pH. Interestingly, intracellular pH is not elevated to ~7.6, but stays at a moderate acidity level, which enhances bacterial survival under total starvation. An intermediate state between full-speed growth and total starvation is the semi-starvation. In semi-starvation, methionine acquisition only comes from synthesis. In this condition, intracellular methionine level is lower than that with sufficient methionine supply, therefore intracellular acidification is still induced, just with a lighter degree. This intracellular acidification also induces the uptake of glutamine. With excessive glutamine supply, intracellular glutamine is deaminated to elevate intracellular pH. Particularly, intracellular pH can be elevated to ~7.6, thus recovering bacterial growth under semi-starvation to the growth with full nutrients.

Among the 20 amino acids, glutamine concentration is highest in human plasma and skeletal muscles (Bergstrom et al., 1974; Lacey and Wilmore, 1990; Kim, 2011). Glutamine concentration is 0.57 ± 0.088 mmol/L (~83 μg/mL) in human plasma. However, methionine concentration is 0.02 ± 0.005 mmol/L (~3 μg/mL) in human plasma, only 3.5% of glutamine. This makes methionine become the least abundant amino acid in human plasma (Bergstrom et al., 1974). In addition, methionine is the one of the 8 essential amino acids for human (Rose, 1949). *S. pneumoniae* can cause septicemia (Yuste et al., 2005). Growth of *S. pneumoniae* in the blood is important for causing septicemia. The low concentration of methionine in blood may cause semi-starvation to *S. pneumoniae*. Cysteine concentration in human plasma is 0.11 ± 0.086 mmol/L (~13 μg/mL), which can provide the substrate for pneumococcal methionine synthesis. The much higher concentration of glutamine in blood can enhance bacterial growth under this semi-starvation. To block the growth enhancement by glutamine, we can develop the drug that targets glutamine transport and deamination in *S. pneumoniae*. This will be a novel strategy to treat pneumococcal disease.

## Materials and methods

### Bacterial cultivation and reagents

The parental strain used in this study is *S. pneumoniae* serotype 2 (D39) (Lanie et al., 2007). Pneumococci were cultured in Todd-Hewitt broth supplemented with 0.5% yeast extract (THY), chemically defined medium (CDM) or tryptic soy agar (TSA) plates with sheep blood (3%) at 37°C, 5% CO_2_ as previously described (Lu et al., 2006). CDM was prepared according to the previous study (Willett and Morse, 1966). Appropriate antibiotics were supplemented to the media when necessary as described (Lu et al., 2006). All chemicals and enzymes for molecular biology were brought from Sigma (Beijing, China) and New England BioLabs (Beijing, China), respectively. All strains used in this study are described in Supplementary Table S1.

### Mutant construction

All gene deletions were operated in strain TH4306, a streptomycin-resistant derivative of strain D39, by natural transformation using Janus cassette (JC)-based counter selection as previously described (Sung et al., 2001; Li et al., 2016). Briefly, the up- and down-stream sequences of target genes and JC were individually amplified. The amplicons were linked by enzymatic digestion and ligation. For natural transformation, the gene was replaced by JC, which contains the kanamycin resistance *kan* gene for selection and *rpsL* for counter-selection. The transformants were selected by kanamycin resistance. For counter-selection, the flanking regions of the target genes were amplified. The amplicons were digested with BsaI and fused as described (Li et al., 2016). The primers used in this work are listed in Supplementary Table S2. The specific setup for construction of each mutant is described in Supplementary Table S3.

### Characterization of bacterial growth and survival

Growth of pneumococci was characterized as previously described (Shelver et al., 2003). Briefly, bacteria were grown in THY to an optical density at 620 nm (OD_620_) of 0.5. Then bacteria were washed twice with Ringer’s solution by centrifugation and resuspension. Bacterial pellets were resuspended in Ringer’s solution to OD_620_ 0.5, and diluted at a 1:100 ratio in standard CDM (Willett and Morse, 1966) or CDM with various modifications in amino acid content. Bacterial growth was measured by determining the value of OD_620_.

### Intracellular pH determination

Intracellular pH was determined using a pH-sensitive green fluorescent protein (pH-GFP) as previously described (Wang et al., 2019; Zhang et al., 2023). Briefly, plasmid pIB166 harboring the pH-GFP gene was transformed into pneumococci by natural transformation. Strains with pH-GFP plasmid were cultured in THY till mid-log phase, then washed and diluted into CDM as in bacterial growth determination. At the time for pH detection, 0.6 OD_620_ bacteria were collected and washed twice by colorless CDM, which excludes glutamine, glutamate, methionine, cysteine, cystine, lysine, Fe_2_SO_4_.7H_2_O, MnSO_4_.4H_2_O and vitamins (Eguchi et al., 2018). Bacterial pellets were resuspended in 1200 μL colorless CDM and dispensed into black 96-well plates (200 μL/well) (Corning Incorporated, United States). Fluorescence was determined at Reading1 (excitation 395 at nm, emission at 510 nm) and Reading2 (excitation at 475 nm, emission at 510 nm) by a microplate reader (BioTek Synergy H1, Agilent, United States). The ratio of “Reading1-Blank1” to “Reading2-Blank2 (X) was used to determining the value of intracellular pH (Y) with an equation: Y = 2.1868 × ln(X) + 7.0626.

### qRT-PCR

Quantitative real-time reverse transcriptase PCR (qRT-PCR) was performed as described (Liu et al., 2017). Briefly, D39 (TH4306) was diluted in duplicate of 7 mL CDM supplemented with various concentrations of methionine and glutamine. At 6 h post inoculation, bacteria were collected and processed for extraction of total RNA using RNAprep Kit (Tiangen, Beijing). Extracted RNA was used to construct cDNA pools with iScript™ cDNA Synthesis Kit (Bio-Rad, United States). The following primer pairs were used to amplify the target genes: Pr15131/Pr15132 (*metE*), Pr17523/Pr17524 (SPD0974), Pr17525/Pr17526 (SPD1296), Pr17527/Pr17628 (SPD1417), Pr17529/Pr17530 (SPD1899), and Pr0023/Pr0024 (*spxB*). SPD0857 (*era*, amplified by Pr7932/Pr7933) was used as a reference gene for normalization of gene expression. The primer sequences are listed in Supplementary Table S2. Each gene was tested using triplicate samples for the first time, and subsequently retested once.

### Protein expression and purification

Expression of glutamine deaminase SPD1296 in *E. coli* BL21(DE3) was achieved using pET28a vector. Briefly, SPD1296 was amplified by Pr18930/Pr18931. Then the amplicon was cloned into BamHI/HindIII site of pET28a and transformed to *E. coli* BL21(DE3). The N-terminal 6 × His tag was added in the construct. *E. coli* BL21(DE3) expressing target proteins was cultured in Luria Broth medium supplemented with 50 μg/mL kanamycin at 37°C. The cells (OD_600_ of 0.5) were induced with 0.2 mM isopropyl-beta-D-thiogalactopyranoside (IPTG) overnight at 16°C before being harvested by centrifugation at 8,000 rpm for 10 min at 4°C and washed once with lysis buffer (50 mM Tris–HCl pH 8.0, 300 mM NaCl and 10 mM imidazole).

For purification, the cells were resuspended in lysis buffer containing 10 μg/mL RNase, 5 μg/mL DNase, 1 mg/mL lysosome and 1 mM phenylmethanesulfonyl fluoride (PMSF) and broken by a French pressure cell at 4°C. The cell debris was removed by centrifugation at 11,000 rpm for 1 h at 4°C. The supernatant was loaded onto a 1 mL Nickel-Sepharose resin column and washed with 30 column volumes (CV) of washing buffer (50 mM Tris–HCl pH 8.0, 300 mM NaCl and 20 mM imidazole). The bound protein was eluted by elution buffer (50 mM Tris–HCl pH 8.0, 300 mM NaCl and 250 mM imidazole) and loaded onto a Superdex 75 gel filtration column equilibrated with ITC buffer (20 mM Na-Mes pH 5.5 and 150 mM NaCl).

### Enzyme activity determination

Glutamine deaminase activity was determined as previously described (Zhang et al., 2019). One mL reaction mixture containing 440 μL glutamine (100 mM), 440 μL Tris–HCl (100 mM, pH 7.5), and 20 μL crude glutaminase was incubated at 37°C for 2 min. Reaction was terminated by adding 100 μL trichloroacetic acid [15% (w/v)]. 200 μL of the reaction mixture was mixed with 800 μL 100% methyl alcohol for protein precipitation. After centrifuged at 12000 rpm, 4°C for 20 min, the supernatant was analyzed by HPLC–MS. Absolute quantification was used to determine the concentration of glutamate. One unit of glutamine deaminase was defined as the amount of enzyme that produces 1 μmol glutamate per minute under the reaction condition. Bradford method with bovine serum albumin was used to determine protein concentration.

### Amino acid quantification

Quantification of intracellular methionine was accomplished by liquid chromatography and mass spectrometry. At 6 h post inoculation in CDM, 0.5 OD_620_ bacteria were collected and washed twice by ice-cold Ringer’s solution by centrifugation at 4°C, 12,000 rpm for 3 min and resuspension. After being frozen by liquid nitrogen, bacterial pellets were resuspended in 1 mL 80% methanol stored at −80°C. The solution was transferred into a 2-ml grinding tube containing 1 g of glass beads (0.4–0.6 mm, BE6098-100 g, EASYBIO, China) and ground by high-throughput tissuelyser (SCIENTZ-48, SCIENTZ, China) with high speed for 10 times (1 min for each time). Between each grinding, there was 1 min for sample cooling in ice water. Ground samples were stored at −80°C for 1 h and then centrifuged at 4°C, 12,000 rpm in a 1.5 mL centrifuge tube for 20 min. The supernatants were collected and dried for methionine quantitation in a vacuum dryer. A 6,500 plus QTrap mass spectrometer (AB SCIEX, United States) coupled with ACQUITY UPLC H-Class system (Waters, United States) was used for metabolite quantitation. Chromatographic separation was achieved using an ACQUITY UPLC BEH Amide column (2.1 × 100 mm, 1.7 μm; Waters). Mobile phase A contained HPLC-grade H_2_O-ACN 5/95 (v/v) with 7.5 mM ammonium formate, and mobile B was H_2_O-ACN 50/50 (v/v) with 7.5 mM ammonium formate. Data were acquired in multiple reaction monitoring (MRM) mode in positive mode. The ion transitions were optimized using chemical standards. The nebulizer gas (Gas1), heater gas (Gas2), and curtain gas were set at 55, 55, and 30 psi, respectively. The ion spray voltage was 5,500 v for positive ion mode. The optimal probe temperature was determined to be 550°C, and the column oven temperature was set at 45°C. SCIEX OS 1.6 software (AB SCIEX, United States) was applied for metabolite identification and peak integration.

### Western blot

Western blot was carried out as previous described (Lu et al., 2008; Liu et al., 2019). Briefly, 1 optical density (OD) D39 bacteria cultured in CDM with different concentrations of methionine and glutamine were washed once with Ringer’s solution by centrifugation and resuspension. Then each bacterial pellet was resuspended in 21 μL lysis buffer (phosphate-buffered saline, 100 mM of dithiothreitol, 25 mM of MgCl_2_, 0.1% Triton-X-100), mixed with 3 μL 100 mM PMSF and 6 μL 5× sodium dodecyl sulfate (SDS)-polyacrylamide gel loading buffer. After boiling for 10 min, 10 μL sample was loaded into the each well of 12% Tris-Glycine SDS-polyacrylamide gel. Proteins were electro-transferred to polyvinylidene difluoride membrane (Shenggong, China). The expression of His_6_-tagged proteins was detected with a mouse anti-His monoclonal antibody (Beyotime Biotechnology, China) at the dilution of 1:1000 and horseradish peroxidase-conjugated goat anti-mouse IgG antibody (Proteintech Group, China) at the dilution of 1:2000. The gray scales of MetE protein were calculated by ImageJ_v1.8.0.

### L-azidohomoalanine (AHA) click labeling

The AHA click labeling assay was carried out as previously described with minor modification (Imami and Yasuda, 2019; Rong et al., 2020). D39 was cultured in CDM with no methionine and 10 or 1,000 μg/mL glutamine. At 6 h post inoculation, 2 OD bacteria were collected and washed twice by Ringer’s solution. Then bacteria were resuspended in 1 mL CDM with no methionine and 100 μg/mL glutamine, which contains 2 mM AHA.HCl. After incubation at 37°C for 30 min, bacteria were washed twice by Ringer’s solution and then resuspended in 50 μL lysis buffer containing 0.5 μL 100× protease cocktail, 100 μM Biotin-Alkyne, 1 mM TCEP, 100 μM THPTA, and 1 mM CuSO_4_. After incubation for 30 min at room temperature, 12.5 μL 5 × SDS loading was added. Samples were then boiled for 10 min. 20 μL sample was loaded into each well of SDS-polyacrylamide gel. The newly synthesized proteins were detected by western blot using HRP-conjugated streptavidin (Proteintech Group, China) at the dilution of 1:2000.

### DNA replication activity determination

DNA replication activity was determined by BeyoClick™ EdU (5-ethynyl-2′-deoxyuridine) cell proliferation kit (Beyotime Biotechnology, China) as previously described with minor modification (Ma et al., 2019). D39 were cultured in CDM with no methionine and 10 or 1,000 μg/mL glutamine. At 6 h post inoculation, 1 OD bacteria were collected and washed twice by Ringer’s solution. Then bacteria were resuspended in 1 mL CDM with 200 μg/mL methionine and 100 μg/mL glutamine, which contains 10 μM Edu. After incubation for 30 min at 37°C, bacteria were treated by fixing solution and permeabilization solution. Then bacterial pellets were incubated with the click reaction solution containing click reaction buffer, CuSO_4_, Azide 488 and click additive solution. After incubation for 30 min at room temperature without light, bacteria were washed and resuspended in Ringer’s solution. After transferring samples into a black 96-well plate (200 μL/well) (Corning Incorporated, United States), fluorescence was determined by a microplate reader (BioTek Synergy H1, Agilent, United States).

### Statistical analysis

All experiments reported in this work were conducted in triplicate samples and repeated at least once. The relevant data are presented as mean ± SEM (standard error of mean), and analyzed by two-tailed unpaired Student’s *t-*test in Graphpad Prism 8. Significant differences are defined by *p-*values of <0.05 (*), <0.01 (**), <0.001 (***), and <0.0001 (****).

## Data availability statement

The original contributions presented in the study are included in the article/Supplementary material, further inquiries can be directed to the corresponding authors.

## Author contributions

CZ: Conceptualization, Data curation, Formal analysis, Investigation, Methodology, Project administration, Resources, Software, Supervision, Validation, Visualization, Writing – original draft, Writing – review & editing. JL: Methodology, Validation, Writing – original draft. XL: Conceptualization, Methodology, Resources, Writing – original draft. YX: Validation, Writing – original draft. QG: Validation, Writing – original draft. QC: Validation, Writing – original draft. WL: Validation, Writing – original draft. XG: Validation, Writing – original draft. SW: Funding acquisition, Writing – original draft.
