# Supplementary material for: Glutamine enhances pneumococcal growth under methionine semi-starvation by elevating intracellular pH
Source: Front Microbiol. 2024 Jul 9;15:1430038. doi: 10.3389/fmicb.2024.1430038 (PMC11263215; doi:10.3389/fmicb.2024.1430038)
Supplement: Supplementary file 1 [file Data_Sheet_1.PDF]

SUPPLEMENTARY MATERIALS

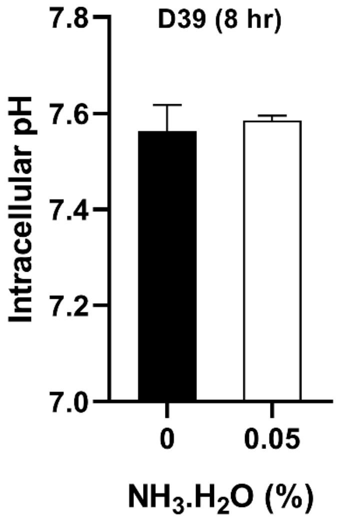

Figure S1. Impact of NH_3 addition on intracellular pH of D39 at 8 hr post inoculation. At 8 hr post inoculation, intracellular pH of D39 cultured in CDM with no or 0.05% NH3.H2O was determined.

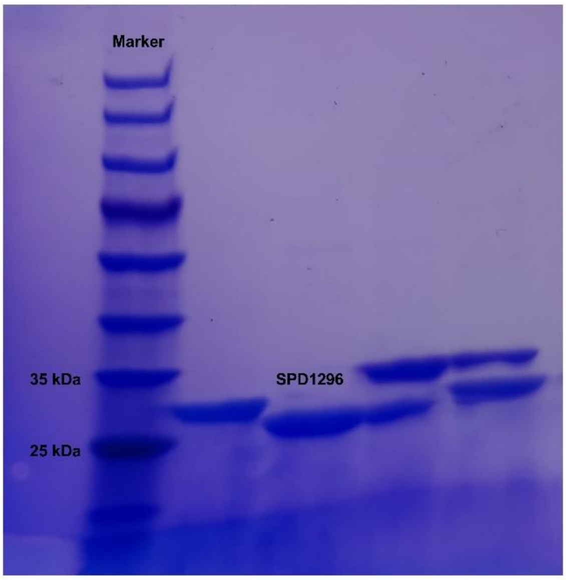

Figure S2. Purified SPD1296 detected by SDS-polyacrylamide gel (SDS-PAGE). After purification, SPD1296 was detected by SDS-PAGE and subsequent Coomassie blue staining. In this figure, there are a marker and four samples. SPD1296 was labeled. The other three samples are not the samples in this paper.

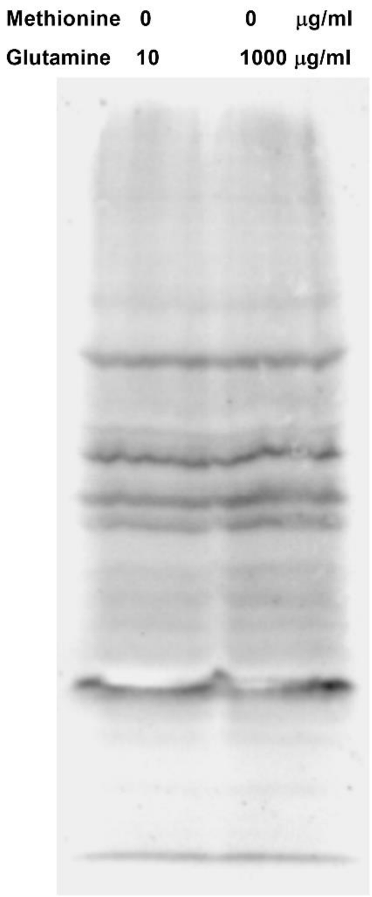

Figure S3. Enhanced translation activity by glutamine supply under methionine semi-starvation. At 6 hr post inoculation, newly synthesized proteins in 2 optical density (OD) D39 cultured with no methionine and 10 μg/ml glutamine or no methionine and 1000 μg/ml glutamine were detected by L-Azidohomoalanine (AHA) click labeling assay.

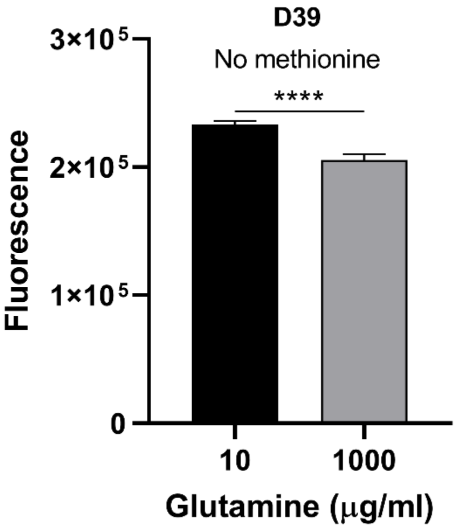

Figure S4. Impact of glutamine on bacterial replication under methionine semi-starvation. At 6 hr post inoculation, replication activity of D39 cultured with no methionine and 10 μg/ml glutamine or no methionine and 1000 μg/ml glutamine was determined by BeyoClick™ EdU (5-ethynyl-2'-deoxyuridine) cell proliferation kit (determining fluorescence).

Table S1. Bacterial strains used in this study

**Strain**	**Description**	**Reference or Source**
TH4306	D39 derivative; *rpsL1*	(1)
LS1	TH4306 derivative; SPD1296 was replaced by JC; TH4306ΔSPD1296::JC	This study
LS2	LS1 derivative; SPD1296 was removed; LS1ΔSPD1296	This study
LS3	TH4306 derivative; plasmid pIB166 with pH-GFP was transformed into TH4306; TH4306 pIB166-pH-GFP	This study
LS4	LS2 derivative; plasmid pIB166 with pH-GFP was transformed into LS2; LS2 pIB166-pH-GFP	This study
LS5	TH4306 derivative; JC was inserted into the upstream of *metE*	This study
LS6	LS5 derivative; JC was replaced by 6 × His tag.	This study

References

1. **Wen ZS, Sertil O, Cheng YX, Zhang SS, Liu X, Wang WC, Zhang JR.** 2015. Sequence Elements Upstream of the Core Promoter Are Necessary for Full Transcription of the Capsule Gene Operon in Streptococcus pneumoniae Strain D39. Infection And Immunity 83:1957-1972.

Table S2. Primers used in this study

Primer number	Sequence (5'-3')
Pr0001	TAACGTGGGAATTTCAACCATTTAC
Pr0002	GAGATCTAGACATGATGTGTCCTCCAAAATTTGTT
Pr0003	GAGACTCGAGTCAACTACAATCATCGGTTTCCCTC
Pr0004	ACGTCACGAACATCACCGAAATAAG
Pr0005	GGTCTCGTGCACCACCACCACCACCACTCAACTACAATCATCGGTTTCCCTC
Pr0006	GGTCTCGTGCATGATGTGTCCTCCAAAATTTGTT
Pr0023	CTTACAAACTTGGTAAACGTCATGC
Pr0024	TTGCACGCCACCATGGAGTTGATTC
Pr7932	GATTGCCATCATGAGTGACAAGG
Pr7933	AGTGTCCACTTCGCGAAGGGT
Pr15131	CAACAAAGAACTCTTTGCGACTGAG
Pr15132	CTTCTTGGATAGCTTCACGTTCTGC
Pr16651	GATGTGCAGAATCCTGAACAGGCTC
Pr16652	GAGATCTAGACTATTTTCCTCGTTCAGCCATGAGA
Pr16653	GAGACTCGAGGATTGAATTTCTCAACTTTTTTACA
Pr16654	TACATGAACCAAGGAATTGACCCAG
Pr16655	GGTCTCGTCCTATTTTCCTCGTTCAGCCATGAGA
Pr16656	GGTCTCGAGGATTGAATTTCTCAACTTTTTTACA
Pr17523	GTTGCCATGGGTGGAACTTTGTACC
Pr17524	TCTGGTGGTGGAAGGAGTTGATATG
Pr17525	GCTGGCTAAGGAAATCACTTCTCAG
Pr17526	AGGTCATTGGAATCTTGCCAACTCC
Pr17527	CTACACGCTCAACCAGACCAATAAC
Pr17528	ATTTCCATAGACAACCTGTCCCAGC
Pr17529	CGATGATTACAATCTGGTCCGTGAC
Pr17530	CTTGCCAGTGACCTTCGATTTCTTG
Pr18930	CGCGGATCCAAAATCGGAATATTGGCCTTGCAAG
Pr18931	CCCAAGCTTTCAACTTTTTTCTTTACACATATTG

Table S3. PCR amplifications used for pneumococcal mutagenesis in this study

Strain ID	Primers		Digestion	Template DNA	Parent strains
	Upstream	Downstream			
LS1	Pr16651/Pr16652	Pr16653/Pr16654	XbaI/XhoI	TH4306	TH4306
LS2	Pr16651/Pr16655	Pr16656/Pr16654	BsaI/BsaI	TH4306	LS1
LS5	Pr0001/Pr0002	Pr0003/Pr0004	XbaI/XhoI	TH4306	TH4306
LS6	Pr0001/Pr0006	Pr0005/Pr0004	XbaI/XhoI	TH4306	LS5
